# A study on dispersion and characterisation of α-mangostin loaded pH sensitive microgel systems

**DOI:** 10.1186/1752-153X-7-85

**Published:** 2013-05-16

**Authors:** Madihah Ahmad, Bohari M Yamin, Azwan Mat Lazim

**Affiliations:** 1 School of Chemical Sciences & Food Technology, Faculty Science and Technology, Universiti Kebangsaan Malaysia, Bangi, Selangor 43600, Malaysia

**Keywords:** α-Mangostin, Microgel, Dynamic light scattering (DLS), Transmission electron microscope (TEM)

## Abstract

**Background:**

α-Mangostin was extracted with methanol from the rind of mangosteen fruit and purified by using silica gel column chromatography technique. The compound is characterised using infrared, ^13^C and ^1^H NMR as well as UV–vis spectroscopy. The α-mangostin dispersion in colloidal systems was studied by incorporating it with an ionic microgel, poly (*N*-Isopropylacrylamide)-co-2VP at different pH.

**Result:**

The DLS result showed the size of microgel-α-mangostin mixture declined from 548 nm to 200 nm upon the increment of the pH. Moreover, it was found the morphology of loaded compound depended largely on the nature of the continuous phase of the microgel system. Interestingly, by manipulating the pH, α-mangostin tends to form crystal at extremely low pH and transforms into spherical shapes at pH 6.

**Conclusion:**

This research shows different structures of the α-mangostin particle that are attributed by adjusting the pH using microgel systems as a template.

## Background

Mangosteen, the ‘queen’ of all fruits is a plant native to Southeast Asia that is used as traditional remedy to treating skin infections wound, improve muscle and bone pain, eating disorder, diarrhea and accelerating wound healing [1,2]. Among the essential phytonutrients found in the rind of the mangosteen, α-mangostin or *1*,*3*,*6*-*trihydroxy*-*7*-*methoxy*, *2*-*8*-*bis* (*3*-*methyl*-*but*-*2*-*enyl*)-*xanthen*-*9*-*one* stands alone in its impressive benefits. Since it was first discovered by W. Schmid in 1855 [3], this compound has attracted many researchers due to its biological active properties such as antioxidant [4], anti bacteria [5], antifungal [6], anti inflammatory [7], anti cancer [8] and anti tuberculosis [9], therapeutic drugs [10] and also being used as mosquitoes larvicide [11]. Furthermore, it has been commercialised as supplement in food products and natural dyes in fabric industries, which are readily available in the worldwide market. However, its poor solubility in aqueous solution and low oral bioavailability are the limiting factors for many applications. Therefore, it is a challenge to the scientists to solve these problems in order to fully utilise this compound deemed suitable for the human body systems. So far, there is only one reported study on the enhancement of the α-mangostin solubility and oral bioavailability by Aisha et al [12]. on the solid dispersion of α-mangostin in water soluble carriers using polyvinylpyrrolidone (PVP). Although this technique showed an improvement on the dissolution of α-mangostin, its commercial use is still very limited, primarily due to manufacturing difficulties and stability problems [13]. As an alternative, a responsive polymer has being used for α-mangostin dispersions.

Much attention has been given to responsive polymers (smart polymers) due to their response ability to external stimuli such as temperature [14], electrolyte, light and pH [15]. One of them is microgel, a polymer colloid particle with three-dimensional network structure that offers many applications from the viewpoint of drug delivery. It can be manipulated as nanoreactor which controls the size property, from macrometers to nanometers [13-15]. Moreover, this polymer is responsive and having large surface network area enables to incorporate with bio-related molecules. This ‘smart’ system is used for bioactive molecules entrapments including drugs, proteins, carbohydrates and DNA [14-16]. Its applications are not only limited for biomedical purposes but are widely applied for the incorporation of inorganic nanocrystals, quantum dots [16,17], magnetic nanoparticles [18,19], optical imaging for living cells and photodynamic therapy [20].

Ionic microgels are formed when at least one of the co-monomer becomes charged specifically when the pH reaches the pK_a_ of that species. Generally monomer such as 2-vinylpyridine [21], acrylic acid [22] and methacrylic acid [23] are used to produce a pH responsive microgel, which can be synthesized via emulsion polymerisation or surfactant free emulsion polymerisation [24]. Xu et al [25] reported on the utilization of poly (ϵ-caprolactone)-pluronic–poly-(ϵ-caprolactone)-dimethylacrylic (PCFC-DMA) as a vitamin B12 carrier. These modified microgels were discovered to be very sensitive towards pH. They found the vitamin B12 could be released from the microgel faster at pH 7.4 than at pH 12. Wang et al [26]. studied the utilization of *poly* (N-isopropylacrylamide-co-acrylamide) as bleomycin drug carrier. The result showed the releasing rates of bleomycin from the microgel exhibited diffusion control at human body temperature.

Both experiments discussed on the successful of using microgel as a carrier for commercially bioactive molecules yet, there was no attempt of incorporating microgel with any organic bioactive compounds. Therefore, this paper discussed on the characterisations of extracted α-mangostin and the attempt of its dispersion in an ionic microgel system, *poly* (*N*-isopropylacrilamide) PNIPAM with a co-monomer 2-vinylpyridine (PNIPAM-co-2VP). The stability over a range of pH in microgel system is also reported

## Results and discussion

### α-Mangostin characterisation

**Figure 1:**
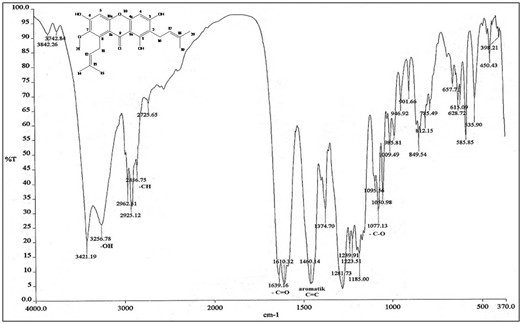
Infra-red spectrum and the structure of α-mangostin.

α-mangostin as in Figure 1 was obtained as a yellow crystalline solid and had a melting point of 175–177°C. The infrared spectrum showed the stretching of hydroxyl (OH) and carbonyl (C = O) group at 3256 and 1639 cm^-1^ respectively. The band at 1460 cm^-1^ showed the presence of aromatic C = C group while the band at 1077 cm^-1^ represents C-O ether bond. The stretching of C-H bond at 2856, 2925 and 2962 cm^-1^ was consistent with the presence of methyl groups in this compound. The infrared spectrum indicated the functional groups in the extracted compound have a similarity to xanthone [27]. NMR (DMSO-d_6_, 400 MHz) δ_H_ (ppm): 1.59 (6H, s, H-14 and H-15),1.70 (6H, s, H19 and H20), 2.50 (4H, d, H11 and H16), 3.18 (3H, s, -OCH_3_), 5.13 (2H, d, H12), 6.33 (1H, s, H4), 6.78 (1H, s, H5), 13.69 (3H, s, C1-OH,C3-OH and C6-OH). ^13^C NMR (DMSO-d_6_, 400 MHz) δ_C_ (ppm): 17.97 (C-14 and C-20), 18.28 (C-15 and C-19), 25.79 (C-11 and C-16), 60.49 (−OCH_3_), 92.54 (C-4), 102.03 (C-5 and C9a), 109.98 (C-2), 110.19 (C-8a), 122.65(C-12), 123.86 (C-17), 130.94 (C-13 and C-18), 136.73 (C-8), 143.63 (C-7), 154.47 (C-4a), 157.20 (C-6), 158.9 (C-10a), 160.11 (C-1), 162.59 (C-3), 181.59 (C-9). The NMR data are consistent with the literature [28].

**Figure 2:**
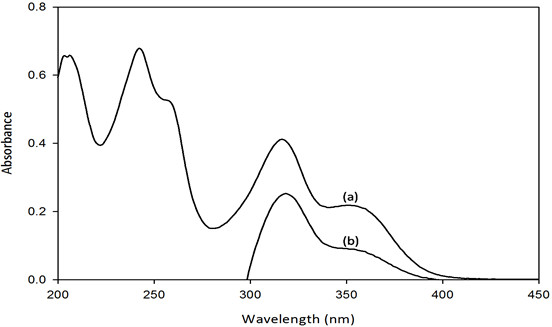
UV–vis spectra of α-mangostin (a) and mixture of PNIPAM-co-2VP/α-mangostin (b).

The UV–vis spectra of α-mangostin as seen in Figure 2 showed maximum absorption peaks at 243, 317 and 352 nm. These values are in agreement with the reported values [28]. Absorption peak at 243 nm represents the C = C chromophore with the excitation energy π → π* transition while the peak at 317 nm is related to C = O chromophore with excitation energy n → π* transition. There is one shoulder at 265 nm indicating the C-O-C with lone pair chromophore denoting n → σ* transition. The existence of shoulder might be due to the interaction of the compound with the protic solvent used. However, when α-mangostin is mixed with PNIPAM-co-2VP microgel, only peaks at 317 and 352 nm are observed while peak at 243 nm and the shoulder disappeared. The absorbance intensity of the mixture peaks is also decreased. This showed an interaction between α-mangostin and the microgel system.

### Incorporating α-mangostin into PNIPAM-co-2VP dispersions

**Figure 3:**
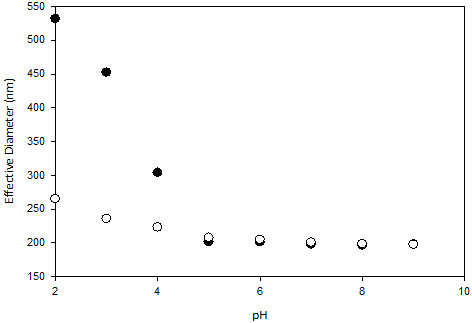
Hydrodynamic diameter of PNIPAM-co-2VP microgel particles with α-mangostin (●) and without α-mangostin (○) as a function of pH measured by DLS.

The hydrodynamic diameter of 0.1 wt% cross-linked PNIPAM-co-P2VP as native microgel particles (without) and with additional α-mangostin (with concentration of 5 x 10^-5^ M) as a function of pH is shown in Figure 3. Under acidic solution conditions, the PNIPAM-co-2VP microgel particles have a large hydrodynamic diameter, however with increasing the pH, their diameter decreases. The largest change in hydrodynamic diameter occurs at approximately pH 4. At low pH, the pyridine groups in the polymer framework are protonated resulting in charged microgel particles. Consequently, it caused electrostatic repulsion between the polymer chains and increased the osmotic pressure within the particles in the network hence led the microgel particles to swell. As a result of the hydrophilic nature of PNIPAM-co-2VP, swelling begins when the pH is a couple of units above the pK_a_ value of 2VP (approximately 4) and shows a gradual swelling profile with decreasing pH. These results are consistent with observations reported by Daly & Saunders [29] and Lazim et. al [30].. Upon adding the α-mangostin, the hydrodynamic diameter profile for microgels decreased nearly half of the original size, which suggests the α-mangostin has infiltrated the networks. In a significant research reported by Lazim et. al [30], if any compounds are most likely to be adsorbed to the surface of the microgel particles and not infiltrated into the microgel network, it would be shown by a fully swollen in hydrodynamic diameter particularly at low pH where positively charged microgels have maximum charge.

In both cases, the particle size reached its optimal de-swollen state at pH 5 (D_h_ ~200 nm). Any further increase in pH had no significant effect on the particle size. The exact mechanism by which the α-mangostin associates with the microgel is still unclear. However, Bradley et.al [31] proposed that the step involved would be the electrostatic interaction between the negatively charged of the additional compound with the cationic groups on the microgel network. This would act to reduce the electrostatic repulsion screening of the charges that would otherwise exists in the network [31].

### TEM imaging

**Figure 4:**
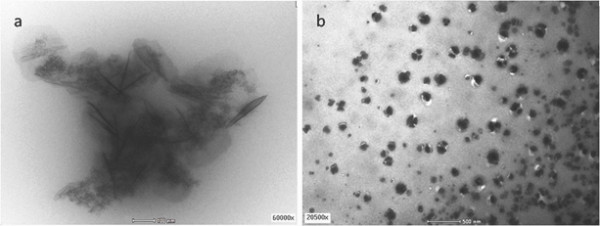
TEM image of mixture α-mangostin/PNIPAM-co-2VP microgel at (a) pH2 and (b) pH6.

The mixtures of microgels and α-mangostin were characterised using the transmission electron microscope (TEM), which allowed a direct morphology dispersion investigation in the systems without staining the necessary process. Figure 4 shows TEM images of up taking α-mangostin into PNIPAM-co-2VP microgel systems at pH 2 and pH 6. It has been established that the microscopic gel systems were used as promising templates to prepare nanoparticles, composite with non-classical shapes of morphology [32,33]. It can be clearly seen that at pH2 α-mangostin showed formation of nanocrystals in PNIPAM-co-2VP microgel systems with average sizes of 148 nm. At low pH, an initially fluid-like system slowed down gradually due to the formation of crystalline structures, hence nucleation occurred homogeneously throughout the systems [34]. Upon increasing the pH by adding the NaOH, the negatively charge concentration increases and neutralization occurs. As a result, an additional attractive force between the microgel particles is introduced [35]resulting the α-mangostin agglomerated. In comparison with acidic ambience, the size of α-mangostin particle at pH 6 is larger with an average size of 217 nm (Figure 4b).

## Conclusion

Yellow compound was successfully extracted from the rind of *Garcinia Mangostana Linn*., which has similarity with α-mangostin characteristics and supported by data that is highly significant with previous literature. The α-mangostin dispersions in microgel systems as a function of pH were investigated. The DLS results showed that there was an interaction in the presence of α-mangostin in PNIPAM-co-2VP microgel systems in swollen state, where the neutralization occurred which then affected the particle sizes. On the contrary, at the collapsed state (pH 6 and higher) there was no contribution to any significant changes to the structure of PNIPAM-co-2VP microgels since it was only a surface interaction. Interestingly, the extreme of pH might not only affect the colloidal dispersion but also the α-mangostin morphology. At pH 2, it resulted as crystal-like shape. However, by increasing the pH value it turned to be agglomerated as big spherical forms. This result showed PNIPAM-co-2VP could be potentially used as a controlled-reactor triggered by pH for α-mangostin particles. Over a variation of pH, interaction of the microgel polymers with distinct crystallographic planes or area of the growing nuclei permits control of the size, shape and structure of the organic compounds. Moreover, it can also be used to overcome its weakness in the aqueous solubility. The future research for investigating the retention, release, the kinetics and its mechanism might be useful for the application of PNIPAM-co-2VP as drug delivery agent for α-mangostin particles to the body system.

## Methods

### Material and instrumentation

All experiments utilized purified water which was milli-Q water standard (PureLab, Elga), with resistivity of 18.2 MΩ cm. Dialysis tubing (Fisher) with a M_w_ cut-off of 12,000-14,000 Daltons was used for microgel purification. For all samples, pH was measured by using a waterproof pH meter (*HI98127*, *pHep Hanna*). For poly (N-isopropylacrylamide) co-2-vinylpyridine (PNIPAM-co 2VP) microgel synthesis, the cross-linking monomers divinylbenzene (DVB, Aldrich, 80%) and N, N-methylenebisacrylamide (BA, Aldrich, 99%), were used without further purification. The initiator used for the cationic microgels was 2, 2’-azobis (2-methylpropionamidine) dihydrochloride (V50, *Waco*, 95%). Aqueous solutions of HCl and NaOH were used to adjust pH. All chemicals and solvents used were reagent grade and used without further purification. Infrared spectra were recorded on a Perkin Elmer GX Spectrometer by using potassium bromide pellet. The size and morphology of the sample was investigated by using Transmission Electron Microscope (TEM) Philips CM12 model.

### UV determination

The Ultra-violet spectra were determined by Shimadzu UV–vis Spectrophotometer (UV 2400PC series). The sample was dissolved in ethanol and the solution was scanned from 200 to 450 nm. Ethanol was used as the background for α-mangostin sample while a mixture of ethanol and pnipam-co-2VP microgel was used for α-mangostin and microgel mixture.

### NMR analysis

The ^1^H and ^13^C nuclear magnetic resonance spectra were measured with Jeol JNM-ECP 400 NMR Spectrometer. Samples were dissolved in dimethylsulfoxide (DMSO)-*d*_*6*_ and chemical shifts were given in parts per million (ppm) relative to tetramethylsilane (TMS) as an internal standard.

### DLS and zeta potential characterisation

Microgel particle sizes and polydispersities index (PDI) were determined by dynamic light scattering (DLS) using a Zetasizer Nano-S (Malvern,PA). The electrophoretic mobility (μ_e_) for α-mangostin and microgel is determined as a function of pH for dispersions at 25°C. The α-mangostin μ_e_ values remain negative across the entire pH range from 2 to 10, whereas the PNIPAM-co-2VP microgel μ_e_ values remain positive which are consistent with the cationic polymer remaining with positive charge.

### Extraction of α-mangostin

Dried mangosteen rind samples were collected from Terengganu, Malaysia and the extraction of α-mangostin was carried out by following the normal procedure of isolating natural products as previously reported [36]. The grinded mangosteen rind was extracted with methanol for three weeks and then separated by column chromatography eluted with the mixture of dichloromethane-hexane (6:4) giving a fine yellow powder.

### Synthesis of PNIPAM-co-2VP microgel

The PNIPAM-co-2VP microgels were synthesized by a surfactant free polymerization technique as previously reported [37]. Briefly, 800 ml of purified water was purged with nitrogen for 30 minutes in a 1 L, five-neck round bottom flask fitted with a mechanical stirrer, which operated at 150 rpm. About 0.50 g cationic initiator 2, 2’-azobis (2-methylpropionamidine) dihydrochloride (V50, *Waco*) was then added to the reaction flask and stirred. In a beaker 200 ml of purified water (milli-Q standard) (PureLab, Elga), 3.75 g of NIPAM and 0.55 g of BA together with 1.25 g of 2-vinylpyridine (2VP) were added together and stirred for 15 minutes. This solution was then added to the reaction vessel with the temperature raised to 70°C. The polymerization reaction was left to proceed for 6 hours with continuous stirring (~150 rpm). The outcome of the dispersion was filtered through glass wool followed by extensive dialysis against milli-Q water for one week with two changes of water per day.

## Competing interest

There is no conflict of interest for all authors of this article.

## Authors’ contributions

MA carried out the extraction, purification and characterization of the compounds as well as conducts the dispersion study. AML carried out the synthesis of microgel and drafting the manuscript. BMY conceived the study, participated in its design and revising the manuscript critically for important intellectual content. All authors read and approved the final manuscript.

## References

[B1] D’Orazio D Raederstorff D Schueler G Wang-Schmidt Y Wolfram S Novel use of organic compounds Patent US 2009 0221693 A1

[B2] Mahabusarakam W Wiriyachitra P Taylor WC Chemical constituents of garcinia mangostana J Nat Prod 1987 50 474 478 10.1021/np50051a021

[B3] Pedraza-Chaverri J Cárdenas-Rodríguez N Orozco-Ibarra M Pérez-Rojas JM Review- medicinal properties of mangosteen (*Garcinia Mangostana*) Food Chem Toxicol 2008 46 3227 3239 10.1016/j.fct.2008.07.024 18725264

[B4] Suvarnakuta P Chaweerungrat C Devahastin S Effects of drying methods on assay and antioxidant activity of xanthones in mangosteen rind Food Chem 2011 1 240 247

[B5] Nguyen PTM Marquis RE Antimicrobial actions of α-mangostin against oral streptococci Can J Microbiol 2011 3 217 225 21358763 10.1139/W10-122

[B6] Ruchadaporn K Kusuma J Niratcha C Antifungal activity of alpha-mangostin against Candida albicans J Oral Sci 2009 51 401 406 10.2334/josnusd.51.401 19776506

[B7] Chen L Ling-Ling Y Ching-Chiung W Anti-inflammatory activity of mangostins from Garcinia mangostana Food Chem Toxicol 2008 46 688 693 10.1016/j.fct.2007.09.096 18029076

[B8] Matsumoto K Akao Y Ohguchi K Ito T Tanaka T Iinumad M Nozawaa Y Xanthones induce cell-cycle arrest and apoptosis in human colon cancer DLD-1 cells Bioorg Med Chem 2005 13 6064 6069 10.1016/j.bmc.2005.06.065 16112579

[B9] Arunrattiyakorn P Suksamrarn S Suwannasai N Kanzaki H Microbial metabolism of α-mangostin isolated from *Garcinia mangostana L* Phytochemistry 2011 72 730 734 10.1016/j.phytochem.2011.02.007 21377704

[B10] Moffet A Shah P Pharmaceutical and therapeutic composition derived from garcinia mangostana L Plant Patent US 2006 055688 A1

[B11] Lan Q Kim M Use of α-mangostin as mosquito larvicide Patent US 2008 0300300 A1

[B12] Aisha AFA Ismail Z Abu-Salah KM Majid AMSA Solid dispersion of α-mangostin improve its aqueous solubility through self-assembly of nanomicelles J Pharm Sci 2011 101 815 825 22081501 10.1002/jps.22806

[B13] Serajuddin ATM Solid dispersion of poorly water-soluble drugs: early promises, subsequent problems, and recent breakthroughs J Pharm Sci 1999 88 1058 1066 10.1021/js980403l 10514356

[B14] Lusançay G Norvez S Iliopoulos I Temperature-controlled release of catechol dye in thermosensitive phenylboronate-containing copolymers: a quantitative study Eur Polym J 2010 46 1367 1373 10.1016/j.eurpolymj.2010.03.020

[B15] Yin J Dupin D Li J Armes SP Liu S pH-induced deswelling kinetics of sterically stabilized poly(2-vinylpyridine) microgels probed by stopped-flow light scattering Langmuir 2008 24 9334 9340 10.1021/la8014282 18642939

[B16] Hasegawa U Nomura SM Kaul SC Hirano T Akiyoshi K Nanogelquantum dot hybrid nanoparticles for live cell imaging Biochem Biophys Res Commun 2005 331 917 921 10.1016/j.bbrc.2005.03.228 15882965

[B17] Fukui T Kobayashi H Hasegawa U Nagasawa T Akiyoshi K Ishikawa I Intracellular delivery of nanogel-quantum dot hybrid nanoparticles into human periodontal ligament cells Drug Metab Lett 2007 1 131 135 10.2174/187231207780363570 19356032

[B18] Gupta AK Wells S Surface-modified superparamagnetic nanoparticles for drug delivery: preparation, characterization, and cytotoxicity studies IEEE Trans Nanobiosci 2004 3 66 73 10.1109/TNB.2003.820277 15382647

[B19] Chatterjee J Haik Y Chen CJ Size dependent magnetic properties of iron oxide nanoparticles J Magn Magn Mater 2003 257 113 118 10.1016/S0304-8853(02)01066-1

[B20] Das M Sanson N Fava D Kumacheva E Zwitterionic poly(betaine-n-isopropylacrylamide) microgels:properties and applications Langmuir 2007 23 196 201 10.1021/la061596s 17190504

[B21] Loxley A Vincent B Equilibrium and kinetic aspects of the pH-dependent swelling of poly(2-vinylpyridine-co-styrene) microgels Colloid Polym Sci 2007 275 1108 1114

[B22] Zhang J Xu S Kumacheva E Polymer microgels: reactors for semiconductor, metal, and magnetic nanoparticles J Am Chem Soc 2004 126 7908 7914 10.1021/ja031523k 15212539

[B23] Saunders BR Crowther HM Vincent B Poly[(methyl methacrylate)-co-(methacrylic acid)] microgel particles: swelling control using pH, cononsolvency, and osmotic deswelling Macromolecules 1997 30 482 487 10.1021/ma961277f

[B24] Tan JPK Goh CH Tam KC Comparative drug release studies of two cationic drugs from pH-responsive nanogels Eur J Pharm Sci 2007 32 340 348 10.1016/j.ejps.2007.08.010 17950583

[B25] Xu X Fu S Wang K Jia W Guo G Zheng X Dong P Guo Q Qian Z Preparation and characterization of vitamin-12 loaded biodegradable pH-sensitive microgels J Microencapsul 2009 26 642 648 10.3109/02652040802610827 19839800

[B26] Wang Q Zhao Y Yang Y Xu H Yang X Thermosensitive phase behavior and drug release of in situ gelable poly(N-isopropylacrylamide-co-acrylamide) microgels Colloid Polym Sci 2007 285 515 521 10.1007/s00396-006-1592-6

[B27] Boonnak N Karalai C Chantrapromma S Ponglimanont C Fun H Kanjana-Opas A Laphookhieo S Bioactive prenylated xanthones and anthraquinones from*Cratoxylum formosum* ssp. Pruniflorum Tetrahedron 2006 62 8850 8859 10.1016/j.tet.2006.06.003

[B28] Yu L Zhao M Yang B Zhao Q Jiang Y Phenolics from hull of Garcinia mangostana fruit and their antioxidant activities Food Chem 2007 104 176 181 10.1016/j.foodchem.2006.11.018

[B29] Daly E Saunders BR A study of the effect of electrolyte on the swelling and stability of poly (N-isopropylacrylamide) microgel dispersions Langmuir 2000 16 5546 5552 10.1021/la991292o

[B30] Lazim AM Bradley M Eastoe J Trickett K Mohamed A Rogeus SE Recovery of gold nanoparticles using pH-sensitive microgels Soft Matter 2010 6 2050 2055 10.1039/c002511a

[B31] Bradley M Vincent B Warren N Eastoe J Vesperinas A Poly (vinylpyridine) core/poly (N-isopropylacrylamide) shell microgel particles: Their characterization and the uptake and release of an anionic surfactant Langmuir 2008 24 2421 2425 10.1021/la703327v 18294014

[B32] Vimala K Samba Sivudu K Murali Mohan Y Sreedhar B Mohana Raju K Controlled silver nanoparticles synthesis in semi-hydrogel networks of poly(acrylamide) and carbohydrates: a rational methodology for antibacterial application Carbohydr Polym 2009 75 463 471 10.1016/j.carbpol.2008.08.009

[B33] Ballauff M Lu Y “Smart” nanoparticles: preparation, characterization and applications Polymer 2007 48 1815 1823 10.1016/j.polymer.2007.02.004

[B34] Muluneh M Sprakel J Wyss HM Mattsson J Weitz DA Direct visualization of pH-dependent evolution of structure and dynamics in microgel suspensions J Phys Condens Matter 2011 23 50 505101 10.1088/0953-8984/23/50/505101 22040676

[B35] Cho JK Meng Z Lyon LA Breedveld V Tunable attractive and repulsive interactions between pH-responsive microgels Soft Matter 2009 5 3599 3602 10.1039/b912105f

[B36] Pedraza-Chaverrí J Reyes-Fermín LM Nolasco-Amaya EG Orozco-Ibarra M Medina-Campos ON González-Cuahutencos O Rivero-Cruz I Mata R ROS scavenging capacity and neuroprottective effect of α-mangostin against 3-nitropropionicacid in cerebellar granule neurons Exp Toxicol Pathol 2009 61 491 501 10.1016/j.etp.2008.11.002 19108999

[B37] Hall RJ Pinkrah VT Chowdhry BZ Snowden MJ Heteroaggregation studies of mixed cationic co-polymer/anionic homopolymer microgel dispersions Colloids Surf A 2004 233 25 38 10.1016/j.colsurfa.2003.06.004

